# A Treatable Neurometabolic Disorder: Glutaric Aciduria Type 1

**DOI:** 10.1155/2014/256356

**Published:** 2014-01-27

**Authors:** S. Pusti, N. Das, K. Nayek, S. Biswas

**Affiliations:** Department of Paediatrics, R. G. Kar Medical College and Hospital, Khudiram Bose Sarani, Kolkata, West Bengal 700004, India

## Abstract

Glutaric aciduria type 1 (GA-1) is an autosomal recessive disorder of lysine, hydroxylysine, and tryptophan metabolism caused by deficiency of glutaryl-CoA dehydrogenase. It results in the accumulation of 3-hydroxyglutaric and glutaric acid. Affected patients can present with brain atrophy and macrocephaly and with acute dystonia secondary to striatal degeneration in most cases triggered by an intercurrent childhood infection with fever between 6 and 18 months of age. We report two such cases with macrocephaly, typical MRI pictures, and tandem mass spectrometry suggestive of glutaric aciduria type 1.

## 1. Introduction

Glutaric aciduria type I (GA-1) is an autosomal recessive disorder of the degradation of lysine, hydroxylysine, and tryptophan, caused by a defect of the enzyme glutaryl-CoA dehydrogenase [1]. Prevalence of GA-1 is 1 in 56000 [2]. Enzyme deficiency results in an accumulation of glutaric and glutaconic acid, which can be measured by urine testing for organic acids. Glutaric acid has a cytotoxic effect and causes cerebral atrophy and brain damage [3]. It is characterized by macrocephaly at birth or shortly after, dystonia, and many times resembling seizures at the first episode, with degeneration of the caudate and the putamen [4]. Therapy consists in carnitine supplementation to remove glutaric acid, a diet restricted in amino acids capable of producing glutaric acid, and prompt treatment of intercurrent illnesses [5]. Early diagnosis and therapy reduce the risk of acute dystonia in patients with GA-1 [6].

## 2. Case Reports

### 2.1. Case 1

**Figure 1:**
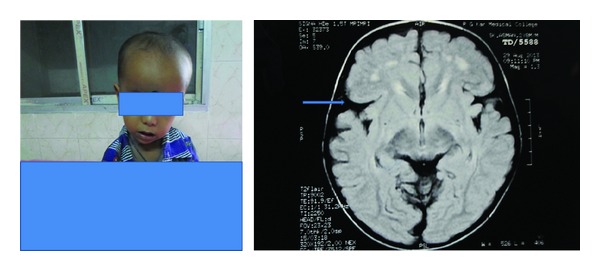
Showing patient with macrocephaly, typical facies, and MRI of his brain reveals frontotemporal atrophy, dilated sylvian fissures with open opercula (arrow), diffuse hyperintense lesions in bilateral basal ganglia, and both frontal white matter and bilateral periventricular area. Widening of the sylvian fissure gives the characteristic “bat-wing” appearance.

A one-and-half-year-old Muslim male child born of consanguineous marriage presented with gross developmental delay and large head size, with the child having history of recurrent episodes of seizures. Although he could smile responsively and feed well, complete head control had not been achieved. On examination he had macrocephaly (OFC 52 cms, expected 47 cms), broad nasal root, hypertelorism, thin sparse hypopigmented hair, and gross developmental delay. Investigations including peripheral blood picture, serum electrolytes, blood glucose, serum ammonia, and liver function test were normal. Urine for tandem mass spectrometry (TMS) report was suggestive of glutaric aciduria. MRI (on T2 weighted MR) brain reveals frontotemporal atrophy, dilated sylvian fissures with open opercula (bat-wing appearance) with hyperintense lesions in bilateral basal ganglia, and the both frontal white matter and bilateral periventricular area suggestive of glutaric aciduria type 1 (Figure 1). Then the child was put on protein restricted diet with carnitine and riboflavin supplementation and anticonvulsant for seizure control. After 6 months of treatment anticonvulsant was withdrawn. After 1 year of followup he remained seizure-free and now he can sit without support, stand with support, and speak disyllable words and head size remained static.

### 2.2. Case 2

**Figure 2:**
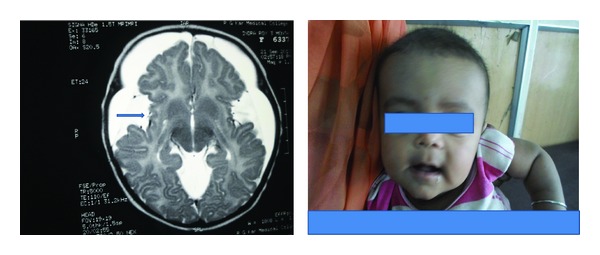
The child with a large head and his MRI brain revealing wide CSF spaces with temporal lobe hypoplasia, bilateral front parietal subdural effusions and dilatation of the sylvian fissures with open opercula (arrow), and high signal intensity seen in bilateral caudate nuclei, putamen, and deep subcortical white matter. Widening of the sylvian fissure gives the characteristic “bat-wing” appearance.

A three-month-old male child born of consanguineous marriage presented with rapid increase in head size. At birth head circumference was 33 cms and showed rapid growth in the last one month and measured 47 cms. Although child could smile responsively and feed well, complete head control had not been achieved. On examination child had macrocephaly, anterior fontanelle bludged, and motor delay. Complete blood picture, serum electrolytes, blood glucose, serum ammonia, liver function test, and CSF study were normal. MRI brain (using T2 weighted FLAIR) reveals wide CSF spaces with temporal lobe hypoplasia, bilateral front parietal subdural effusions, and dilatation of the sylvian fissures with open opercula (bat-wing appearance) and high signal intensity seen in bilateral caudate nuclei, putamen, and deep subcortical white matter suggestive of glutaric aciduria type 1 (Figure 2). The case was confirmed by urinary organic acid analysis by tandem mass spectrometry which revealed a marked excretion of glutaric acid. Then child was put on protein restricted diet with carnitine and riboflavin supplementation. After 3 months of followup child had good head control and could sit with support and head size remained static.

## 3. Discussion

GA-1 is inherited as an autosomal recessive trait and mutations of the GCDH gene on chromosome 19p13.2 [7]. Macrocephaly is a constant feature of GA-1 [1, 7]. The usual age of presentation for GA-1 is 6 months to 2 years of life [7, 8]. Acute neuroregression or dystonia following an initial phase of normal or almost normal development is a common mode of presentation, at times preceded by seizures [7, 8]. Metabolic derangement, which is the hallmark of organic aciduria, is minimal or absent even during acute symptomatic episodes [1]. The other frequent presentation of GA-1 is a chronic encephalopathy associated with choreoathetosis or dystonia [9] often misdiagnosed as athetoid cerebral palsy [7]. GCDH assay from cultured fibroblasts provides the most accurate diagnostic modality [9]. Neuroimaging serves as a useful tool, many times providing the first clue to the diagnosis. MRI is the imaging modality of choice. The earliest feature is frontotemporal atrophy even in the asymptomatic phase but progresses as symptoms develop [1]. Basal ganglia abnormalities, the incomplete opercularization of the insular cortex, widening of the sylvian fissures, and CSF spaces, are characteristic of GA-1 and are encountered in over 90% of patients with this disorder. The early changes in the white matter include delayed myelination and periventricular hypodensities. These are followed by diffuse attenuation and generalized atrophy in the late stages of the disease [1, 7]. The diagnosis of GA-1 rests on the demonstration of urinary excretion of glutaric acid, 3-hydroxyglutaric acid, and glutaconic acid by TMS [1]. The management should include early diagnosis, prevention of acute episodes, and emergency care during intercurrent illnesses, providing a low protein diet restricted in lysine and tryptophan, administration of pharmacological doses of riboflavin, and supplementation of L-carnitine form the mainstay of therapy. Anticonvulsant therapy may be required if seizures are present. Early institution of therapy can provide gratifying results.

## 4. Concussion

GA-1 is an important neurometabolic disorder in children that is sometimes misdiagnosed, but it can be diagnosed easily based on high index of suspicion on typical clinical, neuroimaging, and laboratory findings. Prenatal diagnosis can be made by demonstration of glutaric acid in amniotic fluid [10]. Timely diagnosis and start of treatment are likely to result in a better outcome. It is one of the treatable neurometabolic disorders and, if managed appropriately, favourable prognosis can be given [6]. However, even with treatment, 25 to 35% of children with GA-1 develop some level of motor and intellectual impairment.
